# Temporal Trends and Outcomes in Low-Risk Patients Undergoing TAVI: Insights From the LAPLACE-TAVI Registry

**DOI:** 10.1016/j.jacasi.2026.07.039

**Published:** 2026-09-05

**Authors:** Ryosuke Higuchi, Itaru Takamisawa, Mamoru Nanasato, Shinichiro Doi, Shinya Okazaki, Harutoshi Tamura, Masaki Ishiyama, Hiroaki Yokoyama, Motoki Fukutomi, Shuichiro Takanashi, Mike Saji, Mitsuaki Isobe

**Affiliations:** a Department of Cardiology, Sakakibara Heart Institute, Fuchu, Tokyo, Japan; b Department of Cardiovascular Biology and Medicine, Juntendo University Graduate School of Medicine, Tokyo, Japan; c Department of Cardiology, Pulmonology and Nephrology, Yamagata University Hospital, Yamagata, Japan; d Department of Cardiology, Mie University Hospital, Tsu, Mie, Japan; e Department of Cardiology, Hirosaki University Graduate School of Medicine, Aomori, Japan; f Department of Cardiology, Kawasaki Saiwai Hospital, Kawasaki, Kanagawa, Japan; g Department of Cardiac Surgery, Kawasaki Saiwai Hospital, Kawasaki, Kanagawa, Japan; h Division of Cardiovascular Medicine, Department of Internal Medicine, Toho University Faculty of Medicine, Tokyo, Japan; i Sakakibara Heart Institute, Tokyo, Japan

**Keywords:** eligibility, low-risk, TAVI, trend, volume

## Abstract

**Background:**

Transcatheter aortic valve implantation is now indicated in low-risk patients following favorable outcomes in clinical trials and real-world registries.

**Objectives:**

The authors aimed to investigate temporal trends and long-term outcomes in this population.

**Methods:**

Using a multicenter registry database (2010-2025), we stratified 5,691 patients by their Society of Thoracic Surgeons scores: low risk (<4%, n = 1,580 of 5,691 [27.8%]), intermediate risk (4% to 8%, n = 2,388 of 5,691 [42.0%]), and high risk (>8%, n = 1,723 of 5,691 [30.3%]). Low-risk patients were further classified according to clinical trial eligibility. The primary outcomes were crude 30-day and Kaplan-Meier-estimated 4-year all-cause mortalities.

**Results:**

The annual procedure volume among low-risk patients did not show a sustained increase and declined after peaking in 2022. Low-risk patients were younger, predominantly male, had a higher body mass index (BMI), fewer comorbidities, and fewer procedural complications. They also demonstrated higher rates of elective procedure and transfemoral access. Thirty-day mortality and procedural complication rates remained consistently low among the low-risk cohort; furthermore, 4-year mortality was the lowest among trial-eligible low-risk patients (overall follow-up: 2.2 years). Clinical trial eligibility criteria (frailty [HR: 2.43; 95% CI: 1.43-4.14; *P* = 0.003] and renal dysfunction [HR: 3.00; 95% CI: 1.46-6.20; *P* = 0.010]), female sex (HR: 0.62; 95% CI: 0.44-0.89; *P* = 0.009), BMI (HR: 0.94 per kg/m^2^; 95% CI: 0.90-0.98; *P* = 0.008), and albumin levels (HR: 0.52 per g/dL; 95% CI: 0.36-0.77; *P* = 0.001) were associated with all-cause mortality in low-risk patients.

**Conclusions:**

Despite the overall expansion of transcatheter aortic valve implantation, procedure volume among low-risk patients has not consistently increased. Clinical trial eligibility, female sex, BMI, and albumin levels may refine risk stratification in Society of Thoracic Surgeons–defined low-risk patients. (Outcomes of transcatheter aortic valve implantation in Japanese patients with aortic stenosis: LAPLACE-TAVI registry; UMIN000031133)

In 2002, transcatheter aortic valve implantation (TAVI) was introduced as a treatment for symptomatic high-risk or inoperable patients with severe aortic stenosis (AS). Clinical outcomes following TAVI have improved markedly because of accumulated procedural experience, computed tomography–guided planning, and refinements in bioprosthetic valves and delivery systems.^1^ Several randomized clinical trials involving intermediate- or low-risk patients have demonstrated the noninferiority of TAVI compared with the reference standard of surgical aortic valve replacement (SAVR).^2^, ^3^, ^4^, ^5^ Prompted by these findings, the 2020 American Heart Association/American College of Cardiology guideline recommends TAVI, alongside SAVR, for patients ≥65 years with symptomatic severe AS who are candidates for a transfemoral approach.^6^ Similarly, the 2020 Japanese guideline for the management of valvular heart disease positions TAVI as a viable treatment option across all surgical risk categories.^7^ Generally, TAVI is favored for patients aged ≥80 years, whereas SAVR is preferred for those aged <75 years. For patients of intermediate age (75-79 years), the choice between TAVI and SAVR should be individualized, accounting for comorbidities, procedural feasibility, surgical risk, and patient preferences. Despite the accumulation of evidence from pivotal trials such as the PARTNER 3 (Placement of Aortic Transcatheter Valves) trial and the Evolut Low Risk trial,^4^^,^^5^ as well as subsequent guideline updates^6^, ^7^, ^8^ and regulatory approvals for low-risk patients, detailed real-world data regarding procedural volumes and clinical outcomes in this demographic remain limited.^9^ Because low-risk patients are generally younger and possess fewer comorbidities, they consequently have a longer life expectancy than intermediate- or high-risk patients. Therefore, the lifetime management of AS and the appropriate indication of TAVI in low-risk populations are critical considerations; furthermore, concerns have emerged regarding the potential overuse of TAVI.^10^

Although several risk stratification tools, including the Society of Thoracic Surgeons (STS) score, are widely used to estimate surgical risk, their applicability to TAVI populations remains limited.^11^ To ensure the strict selection of truly low-risk patients, clinical trials have applied additional exclusion parameters beyond the STS score, collectively establishing clinical trial eligibility criteria. Clinical outcomes diverge significantly between patients who meet these eligibility criteria and those who do not.^9^

In this study, we stratified all patients undergoing TAVI who were enrolled in the multicenter LAPLACE-TAVI (aLiAnce for exPloring cLinical prospects of AortiC valvE disease) registry based on their STS risk scores. Specifically, we investigated: 1) the temporal trends regarding procedural volumes, clinical outcomes, and prognostic factors within the low-risk cohort (STS score <4%); and 2) the impact of clinical trial eligibility on mid-term mortality among these low-risk patients.

## Methods

### Study design and participants

This prospective observational study used data from the LAPLACE-TAVI registry (trial number: UMIN000031133). The design and methodology of this registry have been reported previously.^12^^,^^13^ Seven participating hospitals in Japan have prospectively enrolled consecutive patients undergoing TAVI into this registry since April 2010. Indications for TAVI include native severe AS, as well as the structural valve deterioration of surgical bioprostheses or transcatheter aortic valves. We initially identified 5,792 patients who underwent TAVI between April 2010 and May 2025. After excluding 101 patients (1.7%) lacking available STS score, 5,691 patients comprised the final analytic cohort (Supplemental Figure 1). To assess the potential impact of excluding patients with missing STS scores, baseline characteristics and 30-day outcomes were compared between patients included in the primary analysis and those excluded because of missing STS scores (Supplemental Table 1). The study protocol was approved by the institutional review boards of all participating hospitals in accordance with the Declaration of Helsinki. Patients were enrolled in the registry via an opt-out protocol or by providing written informed consent.

### TAVI procedures

Patient selection and procedural strategies, including anesthesia type, access route, device selection, periprocedural antithrombotic therapy, and the necessity of periprocedural coronary revascularization, were determined at the discretions of the local heart team. Generally, indications for intervention and treatment modalities were selected in accordance with contemporary Japanese guidelines.^7^

### STS score and clinical trial eligibility

Patients were stratified according to their STS Predicted Risk of Mortality for isolated SAVR.^14^ Individual STS scores were calculated by the Heart Team before TAVI using the web-based calculator available at the time of procedure. Consistent with previous studies, patients were categorized as low risk (<4%), intermediate risk (4% to 8%), or high risk (>8%).^4^ Beyond STS-based risk stratification, pivotal low-risk trials used several additional exclusion parameters.^4^^,^^5^ For this analysis, we adopted the following 8 of these parameters to define clinical trial eligibility within our low-risk cohort (Supplemental Table 2).

1. bicuspid aortic valve (congenital or acquired)
2. severe mitral regurgitation
3. severe tricuspid regurgitation
4. complex coronary artery disease, defined as left main trunk or 3-vessel disease
5. left ventricular ejection fraction <30%
6. advanced renal dysfunction
7. liver cirrhosis
8. significant frailty or dementia, defined as Clinical Frailty Scale (CFS) >6

Patients classified as low risk based on STS scores were further dichotomized into those who met these clinical trial eligibility criteria and those who did not (Supplemental Figure 1).

### Study outcome

The primary outcome was crude 30-day and Kaplan-Meier estimated 4-year all-cause mortalities. The secondary outcome was 4-year cardiovascular mortality, as estimated via the Kaplan-Meier method. Patients were followed from the date of the index TAVI procedure until death or the last available clinical follow-up, with administrative censoring on June 30, 2025. All clinical events were defined according to the Valve Academic Consortium Criteria-3.^15^

### Statistical analysis

Categorical variables are presented as counts and percentages, whereas continuous variables are expressed as medians with IQRs. Group comparisons were performed using the chi-square test or Fisher exact test for categorical variables, and the Wilcoxon rank-sum test for 2-group comparison or the Kruskal-Wallis test for comparisons among 3 groups for continuous variables. The Kruskal-Wallis test was used to assess overall differences among groups, and no post hoc pairwise comparisons or adjustments for multiple comparisons were performed. Standardized mean differences were calculated to quantify the magnitude of baseline and procedural differences between comparison groups, with larger values indicating greater imbalance. Temporal trends regarding the proportion of low-risk patients and 30-day mortality rates across risk strata were assessed using the Cochran–Armitage trend test. Temporal changes in the proportion of low-risk patients were also evaluated using logistic regression with calendar year modeled as a restricted cubic spline to allow for potential nonlinear temporal changes. All-cause mortality was estimated using the Kaplan-Meier method and compared using the log-rank test. For cardiovascular mortality, cumulative incidence functions were estimated with noncardiovascular death treated as a competing event, and differences between groups were assessed using Gray's test. Univariate and multivariate Cox proportional hazards regression analyses were performed to identify prognostic factors among low-risk patients, incorporating clinically relevant variables selected a priori based on a previous knowledge approach rather than univariate statistical significance.^16^ Multicollinearity among the candidate variables was assessed using variance inflation factors, and the proportional hazards assumption was evaluated using Schoenfeld residuals. The functional form of continuous predictors included in the multivariate Cox model was further assessed using restricted cubic spline analysis. The proportion of missing data for key variables was low, except for the CFS and EuroSCOREⅡ (Supplemental Table 3). Complete-case analysis was primarily used; however, multiple imputation by chained equations was performed for variables with missing data as a sensitivity analysis. A 2-sided *P* value of <0.05 was considered statistically significant. No formal adjustment for multiple testing was performed. Therefore, the results of secondary outcomes and additional analyses should be interpreted with appropriate caution. All analyses were performed using JMP Pro version 19.0.3 (SAS Institute Inc) and R version 4.5.0 (R Foundation for Statistical Computing).

## Results

### Overall cohort

The median age of the overall population was 85 (81-88) years, and 3,643 of 5,691 (64.0%) were female (Supplemental Table 1). Comorbidities included hypertension (n = 4,266 of 5,691; 75.0%), diabetes mellitus (n = 1,362 of 5,688; 23.9%), end-stage renal disease requiring hemodialysis (n = 414 of 5,690; 7.3%), previous stroke (n = 589 of 5,689; 10.4%), chronic obstructive pulmonary disease (n = 478 of 5,594; 8.5%), peripheral artery disease (n = 1,038 of 5,676; 18.3%), and previous myocardial infarction (n = 312 of 5,688; 5.5%). The median STS score was 5.7% (3.8% to 8.9%), and a transfemoral approach was used in 5,281 of 5,628 patients (93.8%). Regarding procedural outcomes, 30-day mortality was 1.3% (95% CI: 1.0%-1.6%) (n = 72 of 5,641), and overt central nervous system injury was observed in 91 of 5,638 patients (1.6%).

### Risk stratification by STS score

The 5,691 patients were stratified into low-risk (n = 1,580 of 5,691 [27.8%]), intermediate-risk (n = 2,388 of 5,691, [42.0%]), and high-risk (n = 1,723 of 5,691 [30.3%]) cohorts based on their STS score (Central Illustration, Supplemental Figure 1). Although annual TAVI procedural volumes increased over time, the proportion of low-risk patients did not demonstrate a consistent increase (*P* for trend = 0.20) and declined in both relative and absolute terms after peaking in 2022 (Central Illustration, Supplemental Figure 2). Restricted cubic spline logistic regression demonstrated a significant association between calendar year and the proportion of low-risk patients (overall *P* < 0.0001), with significant evidence of nonlinearity (*P* for nonlinearity <0.0001) (Supplemental Figure 3). Compared with higher-risk patients, those in the low-risk cohort were younger and less likely to be female; in addition, they had a higher body mass index (BMI) and fewer comorbidities (Table 1). Procedurally, elective interventions and transfemoral access were used more frequently in low-risk patients, who also exhibited lower rates of 30-day mortality and major complications. Furthermore, 30-day mortality within this cohort remained continuously low throughout the study period.

**Central Illustration:**
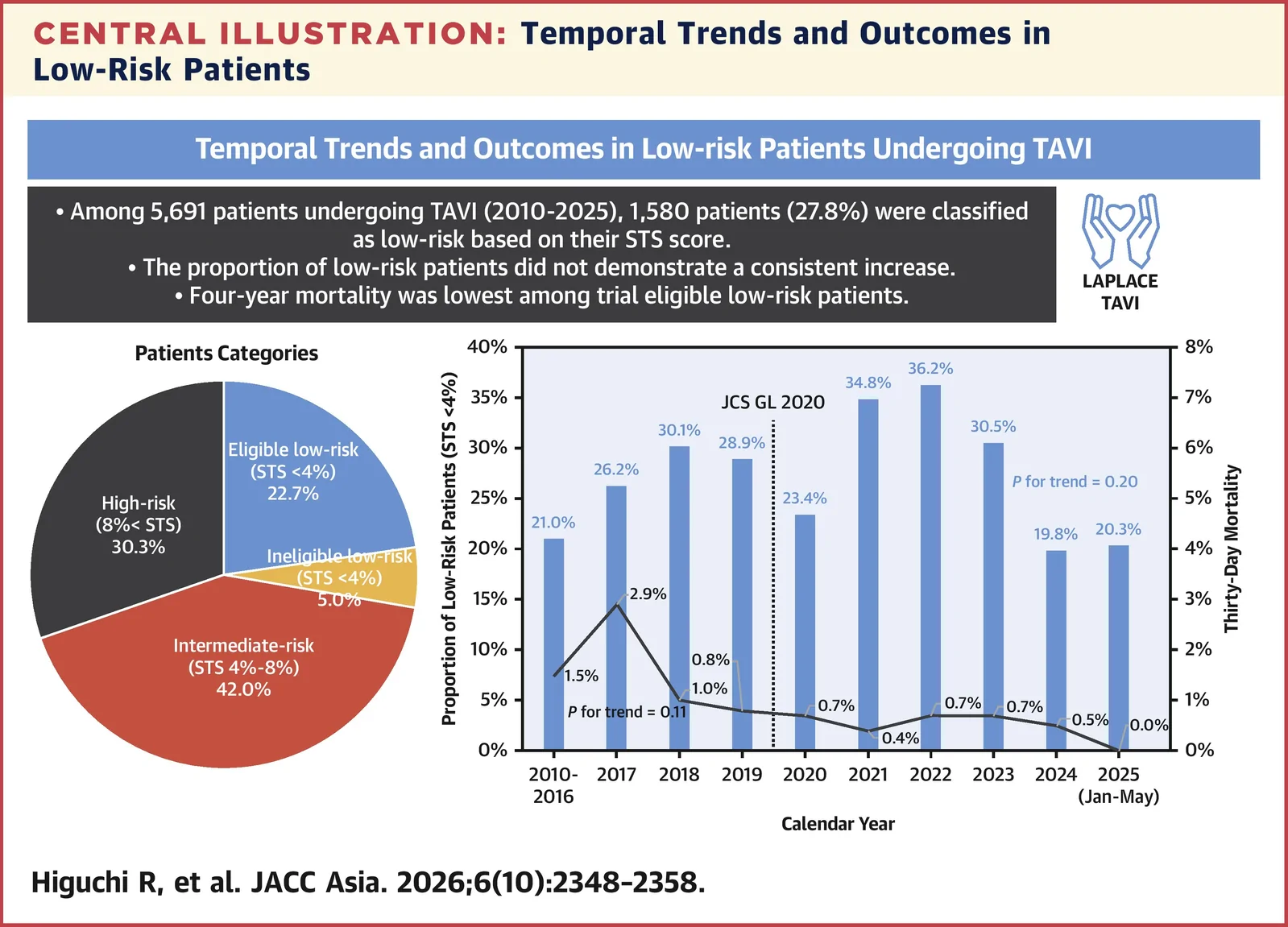
Temporal Trends and Outcomes in Low-Risk Patients The proportion of STS-defined low-risk patients did not consistently increase, and 30-day mortality within this cohort remained low. Four-year mortality was lowest among trial-eligible low-risk patients. JCS GL = Japanese Circulation Society guideline; LAPLACE-TAVI = Outcomes of transcatheter aortic valve implantation in Japanese patients with aortic stenosis; STS = Society of Thoracic Surgeons; TAVI = transcatheter aortic valve implantation.

**Table 1:** Baseline and Procedural Characteristics Among Low- to High-Risk Cohorts

	Low Risk (STS <4%) (n = 1,580)	Intermediate Risk (STS 4%-8%) (n = 2,388)	High Risk (STS ≥8%) (n = 1,723)	*P* Value	SMD
Baseline characteristics					
Age, y	81 (79-84)	85 (82-88)	87 (84-90)	<0.0001	0.676
Female	756 (47.9)	1,664 (69.7)	1,223 (71.0)	<0.0001	0.339
Body mass index, kg/m^2^	23.5 (20.9-25.8)	21.9 (19.6-24.4)	21.1 (19.0-23.5)	<0.0001	0.649
Body surface area, m^2^	1.57 (1.45-1.69)	1.43 (1.32-1.55)	1.40 (1.30-1.50)	<0.0001	0.832
Clinical Frailty Scale	3 (3-4)	4 (3-4)	4 (4-5)	<0.0001	0.471
NYHA functional class Ⅲ/Ⅳ	388 (24.6)	928 (39.0)	978 (56.8)	<0.0001	0.465
Hypertension	1,153 (73.0)	1,830 (76.6)	1,283 (74.5)	0.045	0.070
Diabetes mellitus	366 (23.2)	545 (22.8)	451 (26.2)	0.086	0.067
Hemodialysis	31 (2.0)	107 (4.5)	276 (16.0)	<0.0001	0.348
Stroke	157 (9.9)	242 (10.1)	190 (11.0)	0.53	0.085
COPD	117 (7.5)	195 (8.3)	166 (9.8)	0.018	0.078
Atrial fibrillation	264 (16.7)	552 (23.1)	523 (30.4)	<0.0001	0.254
Carotid artery stenosis	129 (8.2)	168 (7.0)	179 (10.4)	0.004	0.095
Peripheral artery disease	145 (9.2)	394 (16.5)	499 (29.1)	<0.0001	0.357
Previous myocardial infarction	50 (3.2)	119 (5.0)	143 (8.3)	<0.0001	0.149
Hemoglobin, g/dL	12.4 (11.2-13.4)	11.5 (10.4-12.6)	11.1 (10.0-12.2)	<0.0001	0.298
Albumin, g/dL	3.9 (3.7-4.2)	3.8 (3.5-4.1)	3.5 (3.2-3.9)	<0.0001	0.094
eGFR, mL/min/1.73 m^2^	58 (48-70)	51 (40-63)	39 (24-53)	<0.0001	0.669
Ejection fraction, %	64 (60-68)	64 (57-68)	61 (48-67)	<0.0001	0.920
Aortic valve area, cm^2^	0.73 (0.61-0.84)	0.69 (0.55-0.80)	0.66 (0.53-0.80)	<0.0001	0.620
Mean pressure gradient, mm Hg	46 (38-57)	45 (36-58)	41 (31-54)	<0.0001	1.159
Bicuspid aortic valve	110 (7.0)	104 (4.4)	47 (2.7)	0.0007	0.137
EuroSCORE Ⅱ, %	2.8 (1.8-4.1)	4.3 (2.8-6.5)	6.5 (4.0-11.4)	<0.0001	0.662
STS score, %	3.0 (2.4-3.5)	5.6 (4.8-6.6)	11.4 (9.3-15.0)	<0.0001	2.255
Procedural characteristics					
Emergent/Urgent	90 (5.7)	246 (10.3)	374 (21.7)	<0.0001	0.321
Transfemoral approach	1,527 (97.0)	2,226 (93.9)	1,528 (90.8)	<0.0001	0.264
Thirty-day mortality, %	12 (0.8; 95% CI: 0.4-1.3)	13 (0.6; 95% CI: 0.3-0.9)	47 (2.8; 95% CI: 2.1-3.7)	<0.0001	N.A
Procedural complications					
Overt CNS injury	17 (1.1)	35 (1.5)	39 (2.3)	0.074	N.A
Type 3 and 4 bleeding	14 (0.9)	26 (1.1)	23 (1.4)	0.28	N.A
Major vascular complication	20 (1.3)	43 (1.8)	40 (2.4)	0.046	N.A
Acute kidney injury stage 2-4	6 (0.4)	21 (0.9)	29 (1.7)	<0.0001	N.A

Values are n (%) unless otherwise indicated.

CNS = central nervous system; COPD = chronic obstructive pulmonary disease; eGFR = estimated glomerular filtration rate; N.A. = not applicable; SMD = standardized mean difference; STS = Society of Thoracic Surgeons.

### Clinical trial eligibility in low-risk patients

Among low-risk patients, 1,293 of 1,580 (81.8%) met the clinical trial eligibility criteria, whereas 287 of 1,580 (18.2%) did not (Supplemental Figure 1). Reasons for trial ineligibility included a bicuspid aortic valve (n = 110; 7.0%), advanced renal dysfunction (n = 65; 4.1%), significant frailty or dementia (n = 52; 3.3%), and complex coronary artery disease (n = 51; 3.2%) (Supplemental Table 2). Compared with ineligible patients, eligible patients were older and more likely to be female; in addition, they had a higher BMI and a lower prevalence of previous myocardial infarction (Table 2). Estimated glomerular filtration rate (eGFR) and left ventricular ejection fraction were higher in eligible patients, whereas the STS score was comparable between the 2 groups. Regarding procedural outcomes, 30-day mortality was significantly lower among eligible patients.

**Table 2:** Baseline and Procedural Characteristics Among Low-Risk Cohort

	Trial Eligible (n = 1,293)	Trial Ineligible (n = 287)	*P* Value	SMD
Baseline characteristics				
Age, y	82 (79-84)	80 (75-83)	<0.0001	0.362
Female	656 (50.7)	100 (34.8)	<0.0001	0.377
Body mass index, kg/m^2^	23.6 (21.1-26.0)	22.8 (20.6-25.0)	0.003	1.018
Body surface area, m^2^	1.56 (1.45-1.68)	1.60 (1.48-1.71)	0.011	0.674
Clinical Frailty Scale	3 (3-4)	4 (3-6)	<0.0001	0.750
NYHA functional class Ⅲ/Ⅳ	297 (23.0)	91 (31.9)	0.004	0.238
Hypertension	961 (74.4)	190 (66.2)	0.005	0.189
Diabetes mellitus	288 (22.3)	78 (27.2)	0.080	0.120
Hemodialysis	0 (0)	31 (10.8)	<0.0001	0.492
Stroke	120 (9.3)	34 (11.9)	0.19	0.131
COPD	98 (7.7)	19 (6.8)	0.59	0.095
Atrial fibrillation	214 (16.7)	50 (17.9)	0.64	0.182
Carotid artery stenosis	98 (7.6)	30 (10.5)	0.11	0.132
Peripheral artery disease	114 (8.8)	31 (10.9)	0.29	0.097
Previous myocardial infarction	32 (2.5)	18 (6.3)	0.002	0.186
Hemoglobin, g/dL	12.4 (11.3-13.4)	12.3 (11.0-13.4)	0.17	0.082
Albumin, g/dL	3.9 (3.7-4.2)	3.9 (3.5-4.1)	0.0006	0.262
eGFR, mL/min/1.73 m^2^	60 (50-70)	42 (37-67)	<0.0001	0.471
Ejection fraction, %	65 (61-68)	62 (54-66)	<0.0001	1.496
Aortic valve area, cm^2^	0.72 (0.61-0.85)	0.74 (0.60-0.84)	0.93	0.839
Mean pressure gradient, mm Hg	47 (38-57)	45 (36-57)	0.045	1.959
Bicuspid aortic valve	0 (0)	110 (38.3)	<0.0001	1.125
EuroSCORE Ⅱ, %	2.7 (1.8-4.0)	3.3 (1.9-5.3)	<0.0001	0.257
STS score, %	3.0 (2.4-3.5)	3.1 (2.4-3.6)	0.40	0.030
Procedural characteristics				
Emergent/Urgent	69 (5.3)	21 (7.3)	0.20	0.082
Transfemoral approach	1,252 (97.1)	275 (96.5)	0.57	0.169
Thirty-day mortality, %^a^	5 (0.4; 95% CI: 0.2-0.9)	7 (2.5; 95% CI: 1.2-5.0)	0.002	N.A
Procedural complications				
Overt CNS injury	10 (0.8)	6 (2.1)	0.066	N.A
Type 3 and 4 bleeding	10 (0.8)	2 (0.7)	0.90	N.A
Major vascular complication	13 (1.0)	7 (2.5)	0.070	N.A
Acute kidney injury stage 2-4	4 (0.3)	2 (0.7)	0.37	N.A

Values are n (%) unless otherwise indicated.

Abbreviations as in Table 1.

a Detailed causes of 30-day mortality included 2 cardiovascular deaths (1 vascular complication and 1 stroke) and 3 noncardiovascular deaths (2 cases of respiratory failure and 1 bleeding event) in the trial-eligible cohort, and 4 cardiovascular deaths (2 aortic dissections, 1 vascular complication, and 1 stroke) and 3 noncardiovascular deaths (2 cases of respiratory failure and 1 sepsis) in the trial-ineligible cohort.

### Mortality after TAVI

The median follow-up duration for the overall study population was 2.2 years (1.0-4.1 years). Four-year mortality differed significantly across the 3 risk strata and was highest in the high-risk cohort (Figure 1). Following further stratification of the low-risk cohort by clinical trial eligibility, 4-year mortality demonstrated a stepwise increase across the following cohorts: eligible low-risk, intermediate-risk, ineligible low-risk, and high-risk patients (Figure 2). Within the low-risk cohort, clinical trial eligibility status (CFS and eGFR), female sex, BMI, and serum albumin levels were independently associated with all-cause mortality after TAVI (Table 3). All variance inflation factors among candidate predictors were below 3.0, indicating no evidence of substantial multicollinearity (Supplemental Table 4). No significant violation of the proportional hazards assumption was detected in the multivariate Cox model (global test, *P* = 0.11) (Supplemental Table 4, Supplemental Figure 4). Restricted cubic spline analyses showed no significant evidence of nonlinearity for BMI (*P* for nonlinearity = 0.086) or serum albumin levels (*P* for nonlinearity = 0.328), supporting the assumption of linear associations in the multivariate Cox model (Supplemental Figure 5). Results of the sensitivity analysis using multiple imputation were consistent with the primary complete-case analysis. CFS remained independently associated with all-cause mortality (HR: 1.96; 95% CI: 1.19-3.24; *P* = 0.010).

**Figure 1:**
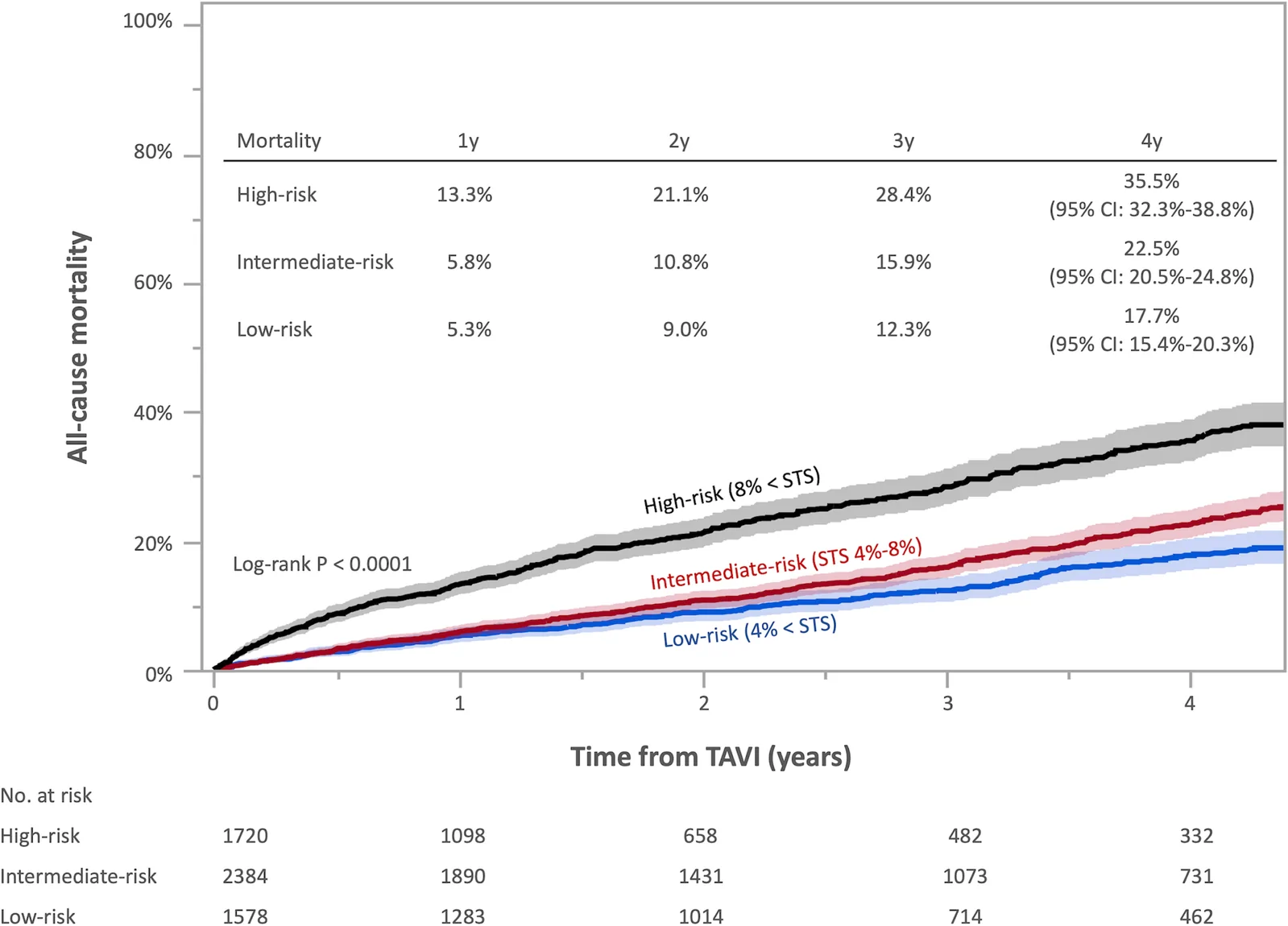
All-Cause Mortality Following TAVI Across Low-, Intermediate-, and High-Risk Cohorts All-cause mortality differed significantly among the 3 risk strata defined by the STS score. Numbers at risk at baseline represents patients with available survival data. STS = Society of Thoracic Surgeons; TAVI = transcatheter aortic valve implantation.

**Figure 2:**
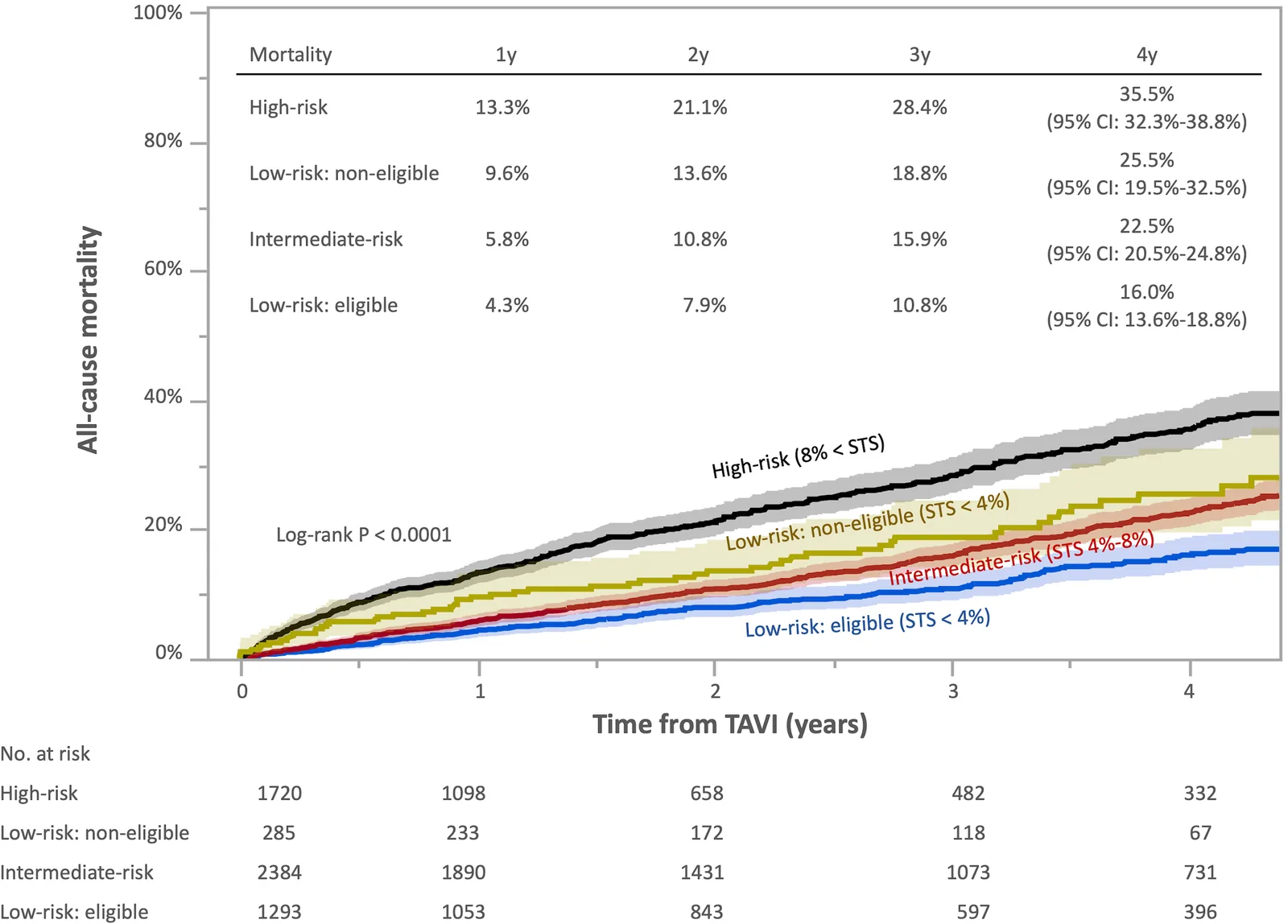
All-Cause Mortality Across Eligible/Ineligible Low-, Intermediate-, and High-Risk Cohorts All-cause mortality was lowest among trial-eligible low-risk patients. Numbers at risk at baseline represents patients with available survival data. Abbreviations as in Figure 1.

**Table 3:** Predictors of Mortality After Transcatheter Aortic Valve Implantation in Low-Risk Patients

	Univariate			Multivariate		
	HR	95% CI	*P* Value	HR	95% CI	*P* Value
Eligibility criteria: exclusion criteria						
Complex CAD: LMT and/or TVD	1.28	0.68-2.41	0.45			
Clinical Frailty Scale ≥6	2.29	1.36-3.87	0.002	2.43	1.43-4.14	0.003
Bicuspid aortic valve	1.24	0.78-1.99	0.36			
eGFR <30 or hemodialysis	2.76	1.36-3.87	0.002	3.00	1.46-6.20	0.010
Other variables						
Age per 1-year increase	1.01	0.99-1.03	0.52			
Female	0.71	0.56-0.91	0.007	0.62	0.44-0.89	0.009
Body mass index per 1-kg/m^2^ increase	0.91	0.88-0.95	<0.0001	0.94	0.90-0.98	0.008
Diabetes mellitus	1.13	0.84-1.51	0.43			
Stroke	1.14	0.78-1.66	0.52			
COPD	0.65	0.37-1.17	0.12			
Atrial fibrillation	1.16	0.84-1.60	0.36			
Carotid artery stenosis	0.94	0.60-1.47	0.78			
Peripheral artery disease	1.41	0.96-2.07	0.094	1.51	0.87-2.60	0.16
Previous myocardial infarction	1.52	0.83-2.79	0.17			
Albumin per 1-g/dL increase	0.41	0.33-0.53	<0.0001	0.52	0.36-0.77	0.001
Transfemoral approach	0.87	0.45-1.66	0.66			

CAD = coronary artery disease; LMT = left main trunk; TVD = triple vessel disease; other abbreviations as in Table 1.

Cardiovascular mortality, which accounted for approximately one-third of overall mortality, was highest in the high-risk group, whereas cardiovascular death rates among the remaining risk strata were similar (Supplemental Figures 6 and 7).

## Discussion

The principal findings of the present study are as follows:

1. Between 2010 and 2025, a total of 5,691 patients who underwent TAVI at 7 centers in Japan were stratified by STS score into low-risk (n = 1,580 [27.8%]), intermediate-risk (n = 2,388 [42.0%]), and high-risk (n = 1,723 [30.3%]) cohorts. The annual procedure volume among low-risk patients did not show a sustained increase and declined after peaking in 2022. Compared with higher-risk patients, low-risk patients were younger and less likely to be female; in addition, they had a higher BMI and fewer comorbidities. From a procedural perspective, elective interventions and transfemoral access routes were used more frequently among low-risk patients; furthermore, 30-day mortality in this group remained consistently low throughout the study period.
2. When the low-risk cohort was further classified by clinical trial eligibility, eligible patients were older and more likely to be female; they also possessed a higher BMI and demonstrated lower rates of previous myocardial infarction, renal dysfunction, and cardiac dysfunction. Moreover, 30-day mortality was lower in the eligible cohort than in the ineligible cohort.
3. Four-year mortality was clearly stratified according to risk category and was lowest among the eligible low-risk cohort. Within the low-risk cohort, clinical trial eligibility (CFS and eGFR), female sex, BMI, and serum albumin levels were identified as independent predictors for all-cause mortality.

### TAVI for low-risk patients

Indications for TAVI have expanded from high-risk or inoperable patients to intermediate-risk and even low-risk populations, driven by improvements in devices, procedural techniques, and clinical outcomes. Landmark trials, including the 1-year outcomes of the PARTNER 3 trial and Evolut Low Risk trial, have supported the recommendation for TAVI in low-risk patients within American, European, and Japanese guidelines.^6^, ^7^, ^8^ In the United States, TAVI has rapidly penetrated the low-risk population, resulting in the treatment of younger patients. Notably, most patients aged 65 to 80 years with severe AS undergo TAVI rather than SAVR.^17^ Japan represents the most rapidly aging society globally, largely owing to increased life expectancy and a declining birth rate.^18^ However, Japanese guidelines remain relatively conservative regarding TAVI indications for patients with severe AS.^7^ The age threshold favoring TAVI is set at 80 years, whereas both SAVR and TAVI are considered viable options for patients aged 75 to 79 years. In the present multicenter study, the proportion of low-risk patients (defined as those with an STS score <4%) did not increase consistently and peaked in 2022. Several potential factors may explain this trend. First, a cautious attitude among both policymakers and the public toward new medical devices and the expansion of clinical indications likely contributes to this pattern. Such structural and cultural factors are known to induce device lag.^19^ Second, the feasibility of repeat interventions following initial TAVI may be more limited among Japanese patients compared with Western populations.^20^, ^21^, ^22^ In our previous study of a subset of the present cohort, post-TAVI computed tomography revealed that approximately two-thirds of patients undergoing initial TAVI were at risk for sinus sequestration on one or both sides during redo TAVI.^20^ This may be partly attributable to the smaller aortic root anatomy in Japanese patients, particularly lower sinus of Valsalva height and sino-tubular junction diameter, which may limit the feasibility of redo TAVI without sinus sequestration or coronary obstruction. Moreover, dedicated leaflet modification tools, such as the ShortCut device, had not been approved in Japan as of April 2026.^23^ Third, clinical outcomes of SAVR remain highly favorable. According to the 2023 annual report of the Japanese Association for Thoracic Surgery, a total of 7,498 SAVR procedures were performed with a 30-day mortality of 1.6%, a figure comparable with that of TAVI (1.2%).^24^

### Clinical trial eligibility and feasibility of redo TAVI

Although the STS score is one of the most widely used tools for surgical risk assessment, its direct applicability is somewhat limited in patients undergoing TAVI, particularly those presenting with risk factors not captured by the metric (eg, frailty).^11^ In pivotal trials evaluating TAVI efficacy in low-risk populations, investigators applied several exclusion parameters beyond the STS score to ensure a homogeneous study population.^4^^,^^5^ Common reasons for screen failure in the PARTNER 3 trial included bicuspid aortic valve, inappropriate annulus size, and unsuitable iliofemoral access, whereas those in the Evolut Low Risk trial were not published. The application of these clinical trial eligibility parameters can significantly influence outcomes in low-risk populations.^9^ For the present study, we established 8 exclusion criteria based on those used in the PARTNER 3 trial and the Evolut Low Risk trial, selected according to their applicability to our database and their clinical relevance based on prior studies^13^^,^^25^, ^26^, ^27^, ^28^, ^29^, ^30^: bicuspid aortic valve, significant mitral or tricuspid regurgitation, complex coronary artery disease, reduced ejection fraction, advanced renal dysfunction, liver cirrhosis, and frailty. Consistent with previous reports, trial-eligible low-risk patients in this study demonstrated the lowest mortality among the stratified cohorts.^9^ The 4-year mortality rate among our trial-eligible patients was 16.0%, a figure substantially higher than the 5-year mortality rates reported in the PARTNER 3 trial and the Evolute Low Risk trial (10.0% and 13.5%, respectively).^31^^,^^32^ This discrepancy may be largely explained by differences in baseline patient age (median 82 years in the present study vs 73.3 years in the PARTNER 3 trial and 74.1 years in the Evolut Low Risk trial). According to the 2023 Japanese life table, the estimated 5-year mortality rates for 74-year-old individuals in the general population are 6.8% for women and 14.8% for men, while the estimated 4-year mortality rates for 82-year-old individuals are 14.2% and 25.5%, respectively, broadly corresponding to those observed in the pivotal trials and the present study.^33^ A survival advantage of female sex has been consistently reported in both the general population and patients who underwent TAVI,^34^ and female sex was associated with lower mortality in the present study. Ikuta et al^35^ reported 10-year outcomes of Japanese patients undergoing TAVI, who corresponded to the intermediate- or high-risk cohorts in the present study, with an acceptable reintervention rate of 4.5% at 10 years. In truly low-risk patients, defined as those meeting all clinical trial-eligibility criteria, life expectancy might exceed the durability of current bioprosthetic valves. Therefore, establishing a lifetime management strategy that accounts for the feasibility of repeat aortic valve intervention is essential.^10^ The valve durability and procedural feasibility of second aortic interventions for both transcatheter and surgical bioprostheses remain critical concerns. The 7-year outcomes of the Evolut Low Risk trial, alongside recent meta-analysis incorporating this trial, suggest that the durability of transcatheter aortic valve may be relatively inferior to that of surgical valves.^36^^,^^37^ The feasibility of redo TAVI may be influenced by individual aortic root anatomy and procedural factors, including valve type, valve size, and implantation depth.^20^, ^21^, ^22^ Consequently, treatment strategies for truly low-risk patients should be determined by integrating the latest clinical evidence with shared decision making.^6^, ^7^, ^8^

### Study limitations

First, the present study is an observational analysis based on a multicenter registry involving 7 hospitals; therefore, it does not represent nationwide data. However, the proportion and temporal trends of low-risk patients in our cohort were consistent with those reported in the annual report of the Japan Transcatheter Valve Therapies, which captures overall TAVI procedural volume in Japan, suggesting that our dataset is reasonably representative.^38^ Second, the predictive accuracy of the STS score in TAVI populations is inherently limited. Because the STS risk calculator is periodically updated, patients receiving an STS score of 4% in earlier versions may present with different clinical profiles than those receiving the identical score in more recent versions.^39^ Consistent with this concern, our restricted cubic spline analysis demonstrated significant temporal changes in the proportion of low-risk patients over time. Nevertheless, a strict 4% threshold was used to define low-risk patients to align with previous studies and maintain external validity.^4^^,^^25^ Third, our database lacked information regarding comparable low-risk patients who underwent SAVR. Specifically, we were unable to determine the proportion of low-risk patients treated with SAVR instead of TAVI, or the clinical rationale underlying treatment selections.

## Conclusions

Among 5,691 patients who underwent TAVI between 2010 and 2025 at 7 Japanese hospitals, 1,580 patients (27.8%) were classified as low risk based on their STS score; notably, the proportion of these patients did not demonstrate a consistent increase over time. Compared with higher-risk cohorts, low-risk patients were younger, and less likely to be female; in addition, they possessed a higher BMI and fewer comorbidities. Procedurally, these patients more frequently underwent elective interventions and transfemoral access, whereas their 30-day mortality remained consistently low. Four-year mortality following TAVI was lowest among trial-eligible low-risk patients. Within the low-risk cohort, clinical trial eligibility status (CFS and eGFR), female sex, BMI, and serum albumin levels were independently associated with all-cause mortality. Establishing a lifetime management strategy is essential, particularly for trial-eligible low-risk patients. Beyond conventional surgical risk scores, additional clinical and anatomical factors should be incorporated to better identify patients at truly low risk and optimize patient selection.

### Data Availability

The data underlying this study are not publicly available due to privacy and ethical restrictions. However, de-identified data may be made available from the corresponding author on reasonable request, subject to approval by the institutional review boards of the participating centers.

### IRB Information

Sakakibara Heart Institute, no. 17-048; Juntendo University Graduate School of Medicine, no. 17-263; Yamagata University Hospital, no. 2019-407; Mie University Hospital, no. H2021 to 049; Hirosaki University Graduate School of Medicine, no. 2020-040; Kawasaki Saiwai Hospital, no. 4-44; Toho University Faculty of Medicine, no. M23087.

## Funding Support and Author Disclosures

Clinical proctor: Dr Takamisawa (Edwards Lifesciences, Medtronic, Abbott Medical), Dr Doi (Medtronic, Abbott Medical), Dr Saji (Medtronic); Screening proctor: Dr Higuchi (Edwards Lifesciences, Medtronic), Dr Doi (Edwards Lifesciences); Lecture fee: Dr Higuchi (Edwards Lifesciences, Medtronic, Abbott Medical), Dr Takamisawa (Edwards Lifesciences, Medtronic, Abbott Medical), Dr Nanasato (Abbott Medical), Dr Saji (Medtronic, Abbott Medical, Daiichi-Sankyo). All other authors have reported that they have no relationships relevant to the contents of this paper to disclose.
