# Supplementary material for: Temporal Trends and Outcomes in Low-Risk Patients Undergoing TAVI: Insights From the LAPLACE-TAVI Registry
Source: JACC Asia. 2026 Sep 5;6(10):2348–58. doi: 10.1016/j.jacasi.2026.07.039 (PMC13654435; doi:10.1016/j.jacasi.2026.07.039)

Supplemental Material

Supplemental Table 1
Supplemental Table 2
Supplemental Table 3
Supplemental Table 4

Supplemental Figure 1. Patient Flow
Among 5,792 patients who underwent TAVI in seven centers between 2010 and 2025, 5,691 patients were included in the analysis after excluding 101 patients without an STS score. Of these, 1,580 (27.8%) patients had an STS score < 4%, and 1,293 (81.8%) were clinical trial eligible.
TAVI = transcatheter aortic valve implantation; STS = Society of Thoracic Surgeons.

Supplemental Figure 2. Procedural Volumes and Proportions across Risk Cohorts
Overall procedural volumes increased over time, whereas the proportion of patients presenting with an STS score < 4% declined after peaking in 2022.
STS = Society of Thoracic Surgeons; TAVI = transcatheter aortic valve implantation; JCS GL = Japanese Circulation Society guideline.

Supplemental Figure 3. Restricted cubic spline analysis showing the association between calendar year and the predicted proportion of low-risk patients.
The solid line represents the predicted proportion. Restricted cubic spline logistic regression demonstrated a significant temporal association (overall $p < 0.0001$) with significant evidence of nonlinearity (P for non-linearity < 0.0001).

Supplemental Figure 4. Schoenfeld residual plots for the multivariate Cox proportional hazards model
The proportional hazards assumption was assessed using Schoenfeld residuals. The x-axis represents follow-up time (days).
eGFR = estimated glomerular filtration ratio

Supplemental Figure 5. Restricted cubic spline analyses of BMI and albumin for all-cause mortality in the multivariate Cox proportional hazards model
Solid lines indicate the estimated hazard ratios, and shaded areas represent 95% confidence intervals. No substantial evidence of nonlinearity was observed for either variable.
BMI = body mass index.

Supplemental Figure 6. Cumulative Incidence Function Curves for Cardiovascular Mortality According to Low-, Intermediate-, and High-risk Cohorts
Cardiovascular mortality was highest in the high-risk cohort, whereas cardiovascular death rates among the remaining risk strata were similar.
TAVI = transcatheter aortic valve implantation; STS = Society of Thoracic Surgeons.

Supplemental Figure 7. Cumulative Incidence Function Curves for Cardiovascular Mortality According to Low- (Eligible and Ineligible), Intermediate-, and High-risk Cohorts
Cardiovascular mortality was highest in the high-risk group, whereas cardiovascular death rates among the remaining risk strata were similar.
TAVI = transcatheter aortic valve implantation; STS = Society of Thoracic Surgeons.

Supplemental Table 1. Baseline and Procedural Characteristics among Overall Patients

	Overall (n = 5,691)	Patients missing STS score (n = 101)	P-value	SMD
**Baseline characteristics**				
Age, years	85 (81-88)	85 (81-88)	0.59	0.030
Female	3,643 (64.0%)	61 (60.4%)	0.94	0.068
Body mass index, kg/m^2	22.0 (19.7-24.6)	22.4 (20.0-25.4)	0.30	0.009
Body surface area, m^2	1.45 (1.33-1.58)	1.46 (1.36-1.59)	0.30	0.044
Clinical Frailty Scale	4 (3-5)	4 (3-4)	0.011	0.37
NYHA class Ⅲ/Ⅳ	2,294 (40.4%)	41 (40.6%)	0.99	0.073
Hypertension	4,266 (75.0%)	76 (75.2%)	0.95	0.041
Diabetes mellitus	1,362 (23.9%)	33 (32.7%)	0.11	0.202
Hemodialysis	414 (7.3%)	9 (8.9%)	0.795	0.066
Stroke	589 (10.4%)	15 (14.9%)	0.978	0.157
COPD	478 (8.5%)	15 (14.9%)	0.14	0.212
Atrial fibrillation	1,339 (23.5%)	22 (21.8%)	0.99	0.249
Carotid artery stenosis	476 (8.4%)	6 (5.9%)	0.87	0.093
Peripheral artery disease	1,038 (18.3%)	29 (28.7%)	0.12	0.093
Previous myocardial infarction	312 (5.5%)	7 (6.9%)	0.662	0.063
Hemoglobin, g/dl	11.6 (10.5-12.7)	11.9 (10.7-12.9)	0.071	0.122
Albumin, g/dL	3.8 (3.4-4.0)	3.7 (3.4-4.0)	0.11	0.020
eGFR, ml/min/1.73m^2	50 (37-63)	55 (38-65)	0.29	0.055
Ejection fraction, %	64 (56-68)	64 (59-69)	0.041	0.212
Aortic valve area, cm^2	0.70 (0.56-0.81)	0.70 (0.59-0.80)	0.57	0.021
Mean pressure gradient, mmHg	44 (35-57)	42 (31-49)	0.011	0.12
Bicuspid aortic valve	261 (4.6%)	3 (3.0%)	0.70	0.069
EuroSCORE Ⅱ, %	4.1 (2.6-6.7)	5.2 (3.2-6.9)	0.20	0.224
STS score, %	5.7 (3.8-8.9)	-	-	-
**Procedural characteristics**				
Emergent/Urgent	710 (12.5%)	2 (2.0%)	0.003	0.413
Transfemoral approach	5,281 (93.8%)	90 (89.1%)	0.38	0.100
Thirty-day mortality, %	72 (1.3%; 95% CI: 1.0%-1.6%)	1 (1.0%; 95% CI: 0.03%-5.5%)	0.99	0.046
Procedural complications				
Overt CNS injury	91 (1.6%)	2 (2.0%)	1.00	0.068
Type 3 & 4 bleeding	63 (1.1%)	0 (0%)	0.95	0.152
Major vascular complication	103 (1.8%)	0 (0%)	0.59	0.196
Acute kidney injury stage 2-4	56 (1.0%)	0 (0%)	0.79	0.146

STS = Society of Thoracic Surgeons; SMD = standardized mean difference, NYHA = New York Heart Association; COPD = chronic obstructive pulmonary disease; eGFR = estimated glomerular filtration rate; CI = confidence interval; CNS = central nervous system.

Supplemental Table 2. Eligibility for Low-risk Trials

	PARTNER 3 trial	Evolut Low Risk trial	Present study*
**Inclusion criteria**			
STS score	< 4%	< 3%	< 4%
**Major exclusion criteria**			
Unfeasible iliofemoral vessel	+	+	
Bicuspid aortic valve	+	+	+: 110 (7.0%)
Severe mitral regurgitation	+	+	+: 3 (0.2%)
Moderate or severe mitral stenosis		+	
Severe tricuspid regurgitation		+	+: 2 (0.1%)
Pre-existing any prosthetic heart valve	+	+	
Complex coronary artery disease	+	+	+: 51 (3.2%)
Symptomatic carotid artery disease	+	+	
Hypertrophic obstructive cardiomyopathy	+	+	
Ejection fraction < 30%	+		+: 13 (0.8%)
eGFR < 30 ml/min/1.73m^2 and/or RRT	+		+: 65 (4.1%)
Severe lung disease	+		
Severe pulmonary hypertension	+		
Liver cirrhosis	+		+: 19 (1.2%)
Significant frailty or dementia	+	+	+: 52 (3.3%)
Significant aortopathy	+	+	
Body mass index > 50 kg/m^2	+		
Limited life expectancy (< 24 months)	+	+	

PARTNER = Placement of Aortic Transcatheter Valves; STS = Society of Thoracic Surgeons; eGFR = estimated glomerular filtration rate; RRT = renal replacement therapy.
*The number with percentage indicated patients fulfilling respective exclusion criteria among low-risk cohort defined as STS score < 4%.

Supplemental Table-3. Proportion of Missing Data among Overall Cohorts (n = 5,691)

	Missing data
**Baseline characteristics**	
Age, years	0 (0%)
Female	0 (0%)
Body mass index, kg/m^2	12 (0.2%)
Body surface area, m^2	83 (1.5%)
Clinical Frailty Scale	1,854 (32.6%)
NYHA class Ⅲ/Ⅳ	11 (1.9%)
Hypertension	0 (0%)
Diabetes mellitus	3 (0.05%)
Hemodialysis	1 (0.02%)
Stroke	2 (0.04%)
COPD	97 (1.7%)
Atrial fibrillation	3 (0.05%)
Carotid artery stenosis	2 (0.04%)
Peripheral artery disease	15 (0.3%)
Previous myocardial infarction	3 (0.05%)
Hemoglobin, g/dl	5 (0.09%)
Albumin, g/dL	52 (0.9%)
eGFR, ml/min/1.73m^2	22 (0.4%)
Ejection fraction, %	18 (0.3%)
Aortic valve area, cm^2	72 (1.3%)
Mean pressure gradient, mmHg	20 (0.4%)
Bicuspid aortic valve	18 (0.3%)
EuroSCORE Ⅱ, %	916 (16.1%)
STS score, %	0 (0%)
**Procedural characteristics**	
Emergent/Urgent	9 (0.2%)
Transfemoral approach	63 (1.1%)
Thirty-day mortality, %	50 (0.9%)
Procedural complications	
Overt CNS injury	53 (0.9%)
Type 3 & 4 bleeding	56 (1.0%)
Major vascular complication	57 (1.0%)
Acute kidney injury stage 2-4	59 (1.0%)

NYHA = New York Heart Association; COPD = chronic obstructive pulmonary disease; eGFR, estimated glomerular filtration rate; STS = Society of Thoracic Surgeons; CNS = central nervous system.

Supplemental Table 4. Diagnostics of the Multivariate Cox Proportional Hazards Model

	VIF	PH test (P-value)
**Eligibility criteria: exclusion criteria**		
Complex CAD: LMT and/or TVD	1.07	-
Clinical Frailty Scale ≥ 6	1.04	0.53
Bicuspid aortic valve	1.04	-
eGFR < 30 or hemodialysis	1.02	0.48
**Other variables**		
Age	1.68	-
Female	1.08	0.27
Body mass index	1.03	0.012
Diabetes mellitus	1.07	-
Stroke	1.03	-
COPD	1.06	-
Atrial fibrillation	1.03	-
Carotid artery stenosis	1.04	-
Peripheral artery disease	1.07	0.46
Previous myocardial infarction	1.13	-
Albumin	1.05	0.25
Transfemoral approach	2.96	-
Global	-	0.11

VIF = variance inflation factor; PH, proportional hazards; CAD = coronary artery disease; LMT = left main trunk; TVD = triple vessel disease; eGFR = estimated glomerular filtration ratio; COPD = chronic obstructive pulmonary disease.

Supplemental Figure 1

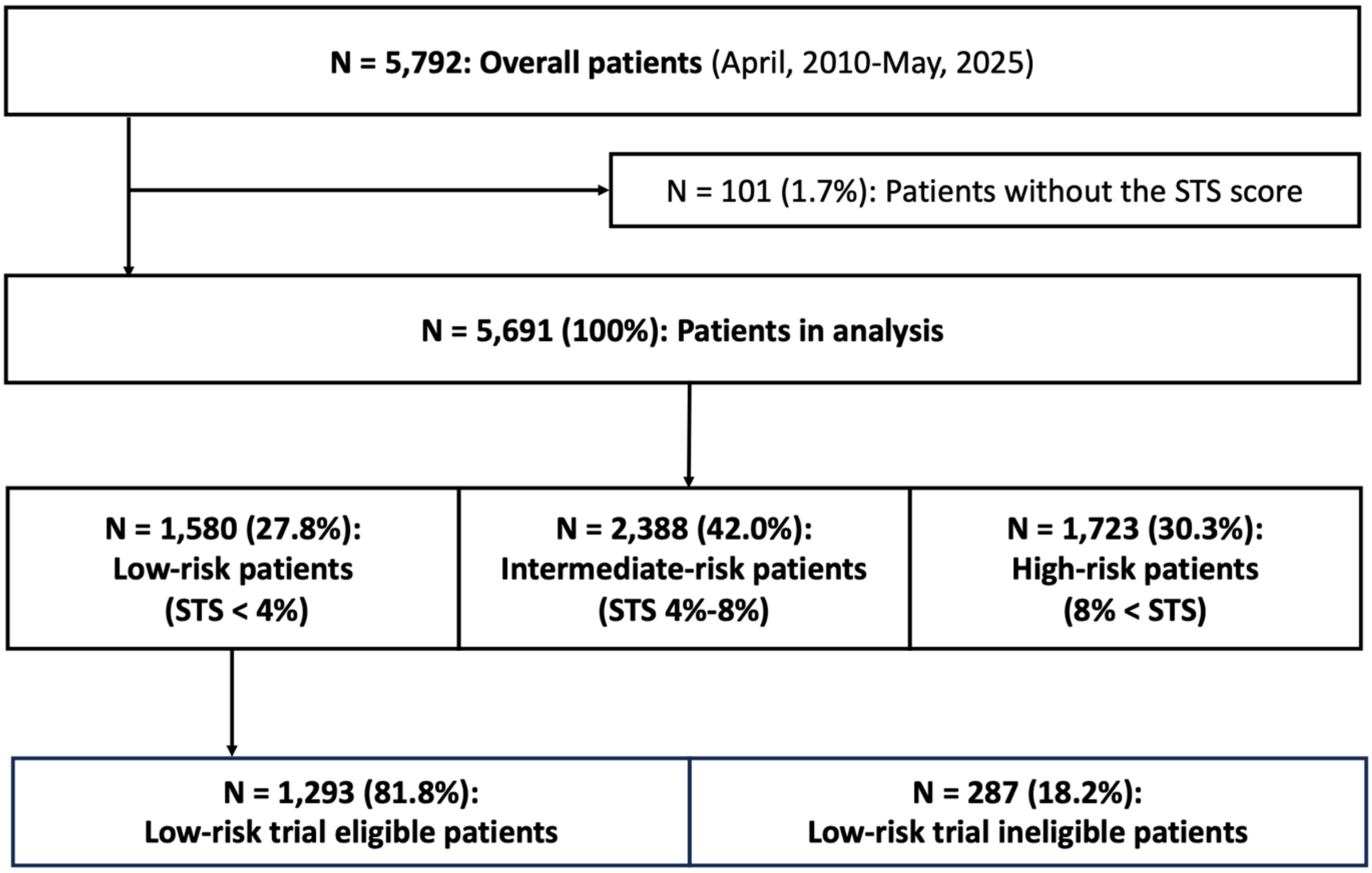

Supplemental Figure 2

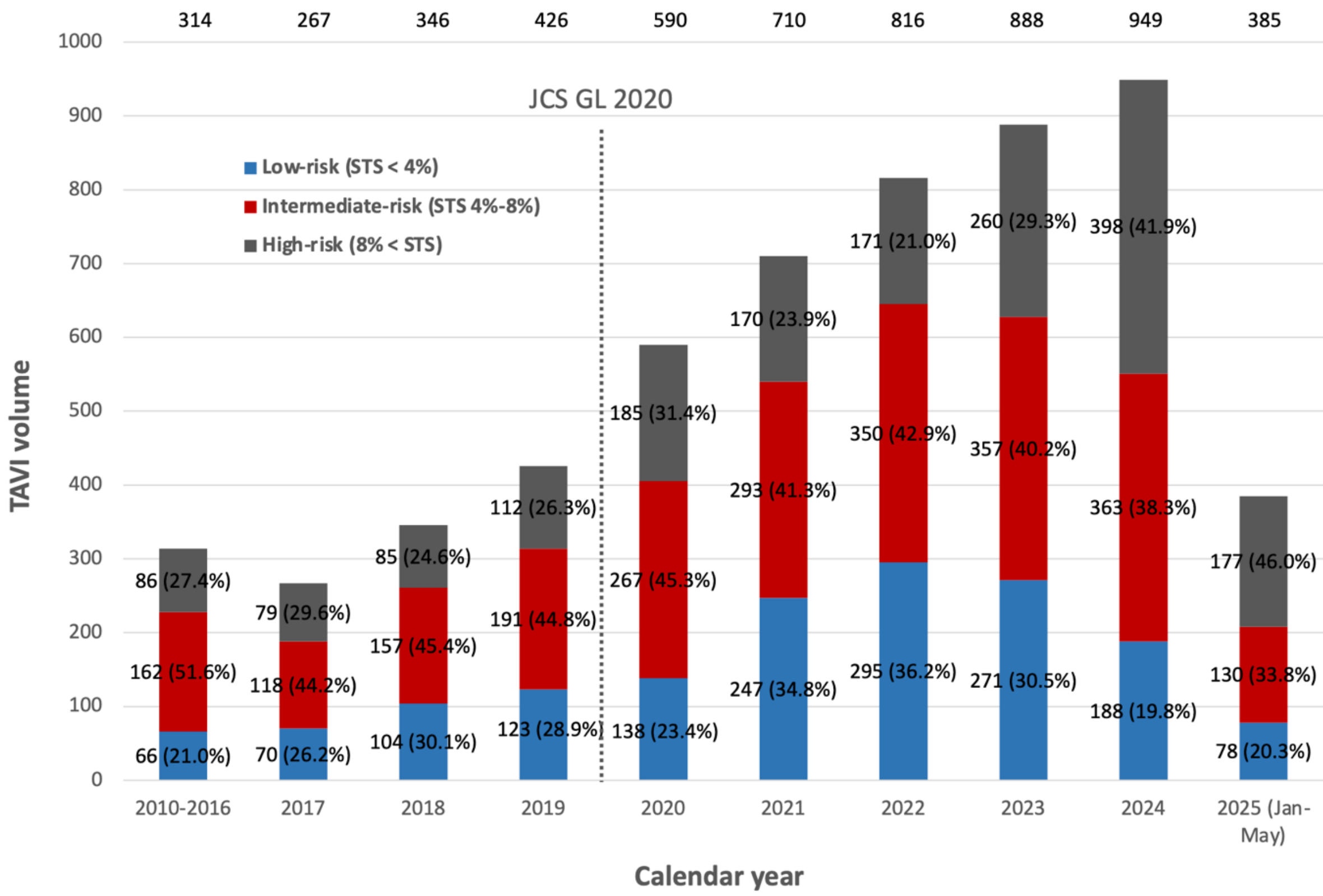

Supplemental Figure 3

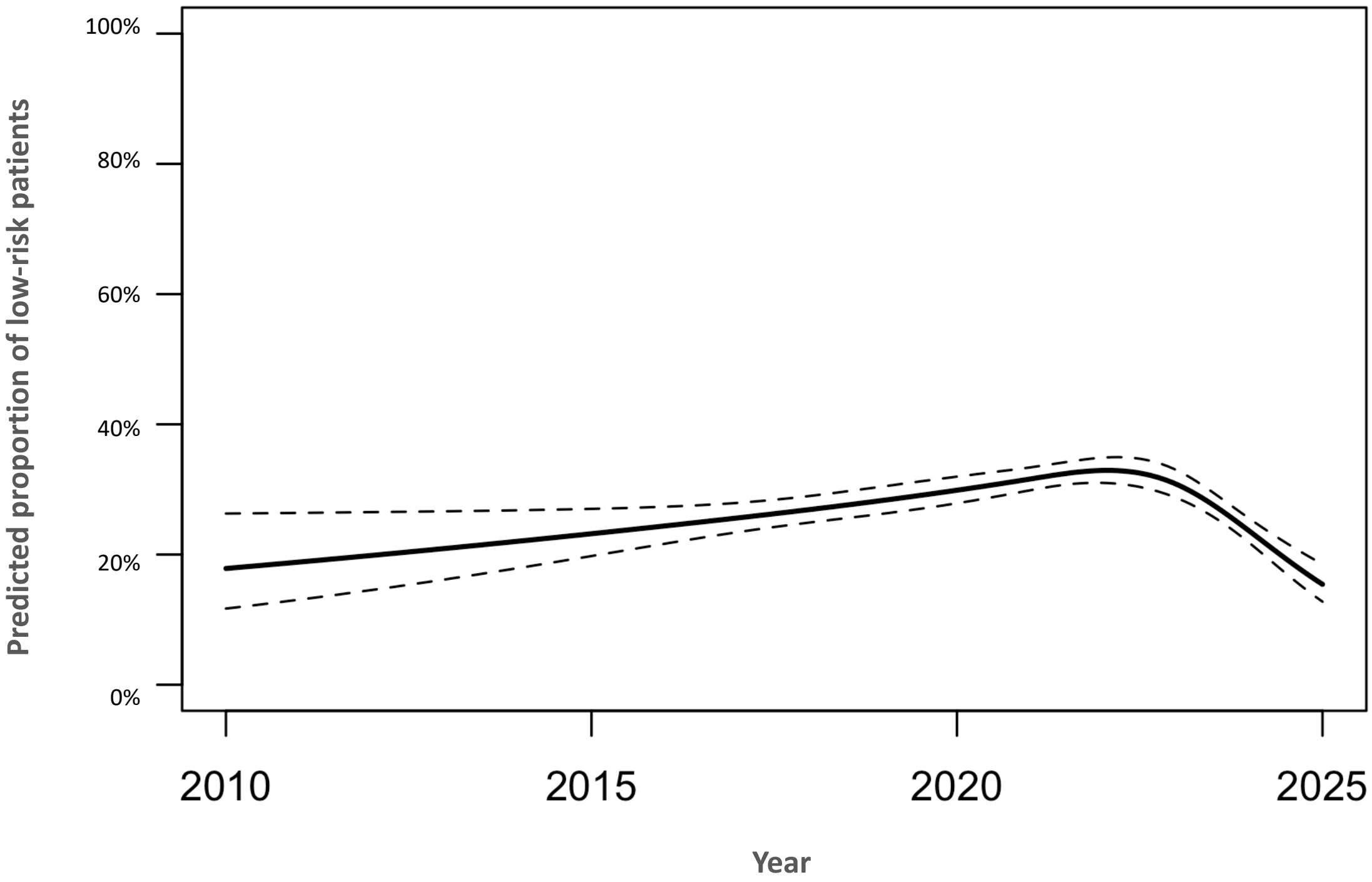

Supplemental Figure 4

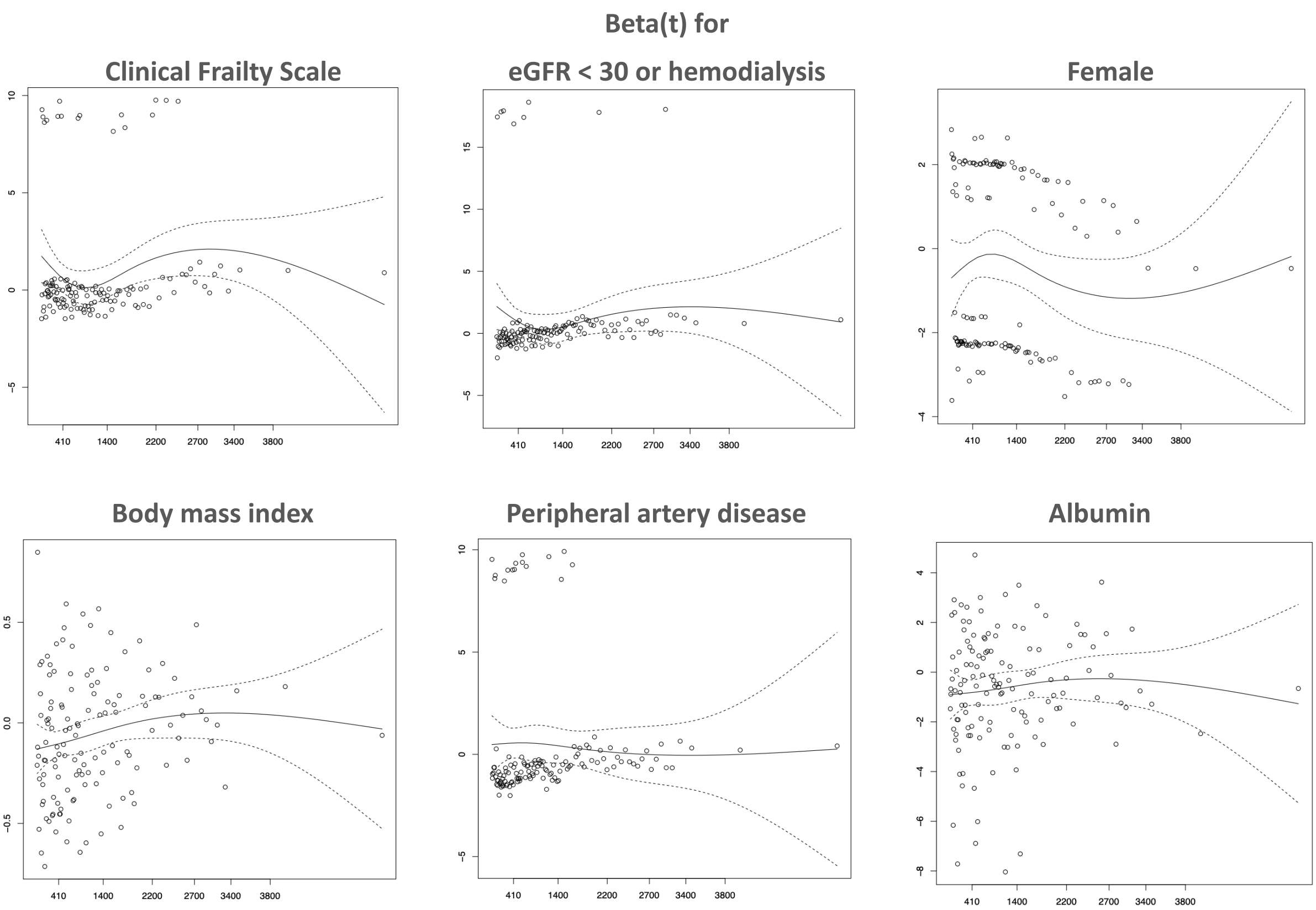

Supplemental Figure 5

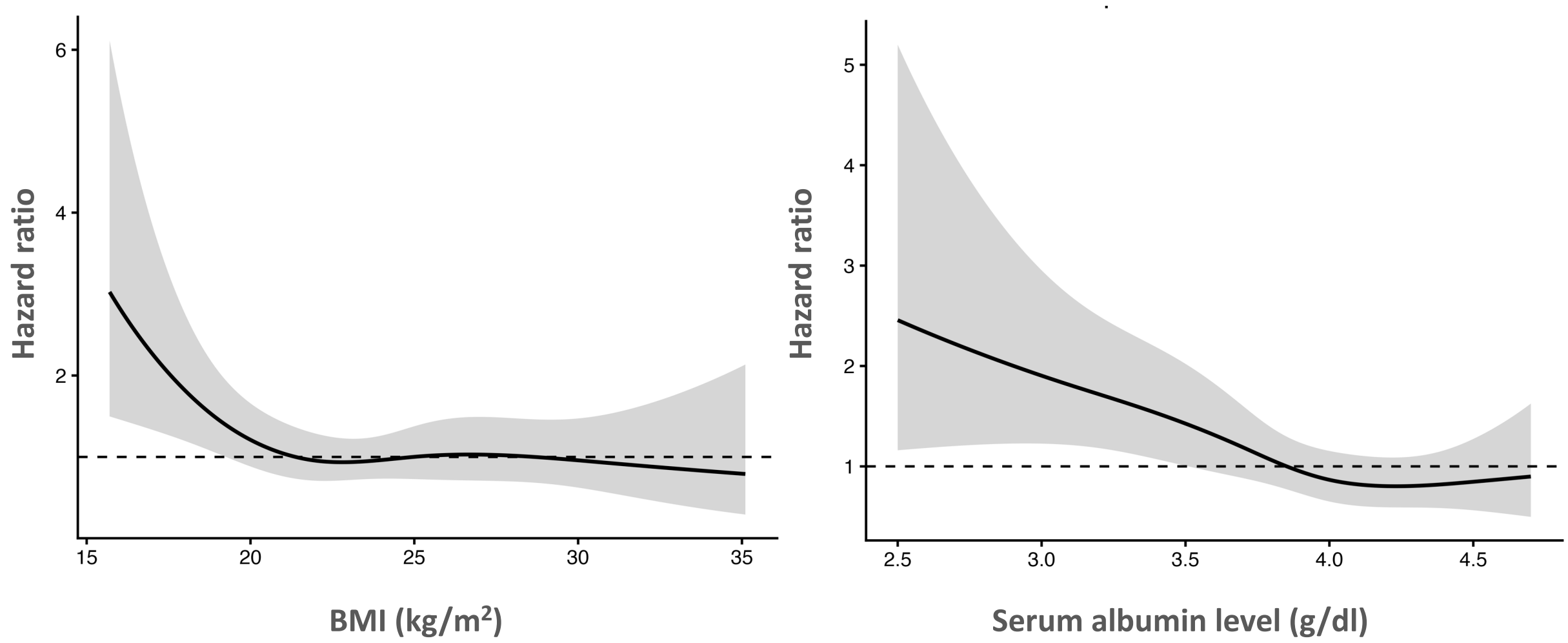

Supplemental Figure 6

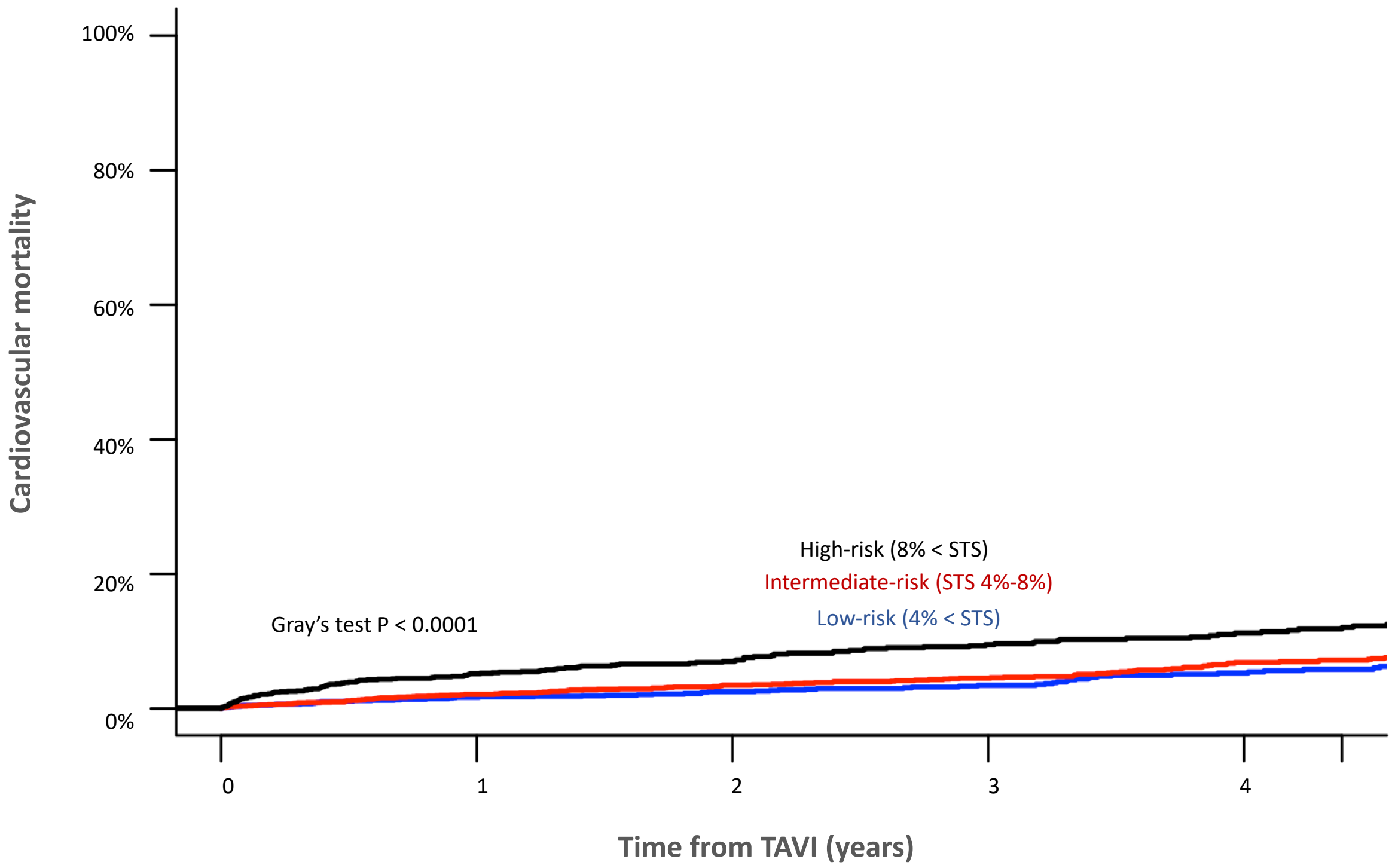

Supplemental Figure 7

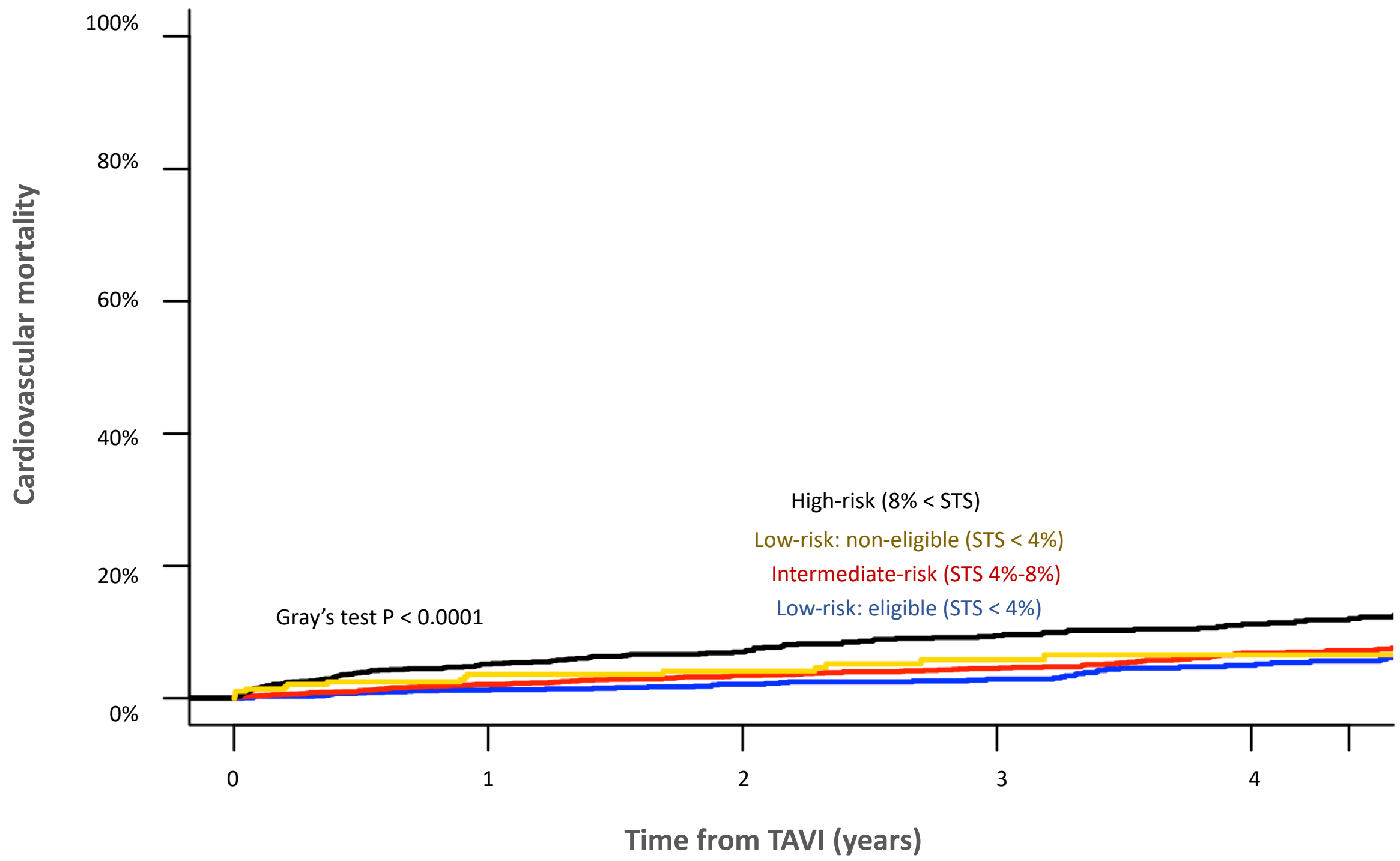

Supplement: Supplementary file 1 [file mmc1.pdf]
